# Supplementary material for: Exercise enhances skeletal muscle regeneration by promoting senescence in fibro-adipogenic progenitors
Source: Nat Commun. 2020 Feb 14;11:889. doi: 10.1038/s41467-020-14734-x (PMC7021787; doi:10.1038/s41467-020-14734-x)
Supplement: Supplementary file 1 — Supplementary Information [file 41467_2020_14734_MOESM1_ESM.pdf]

Supplementary Information for

Exercise enhances skeletal muscle regeneration by promoting senescence in fibro-adipogenic progenitors

Saito *et al.*

Supplementary Figure 1

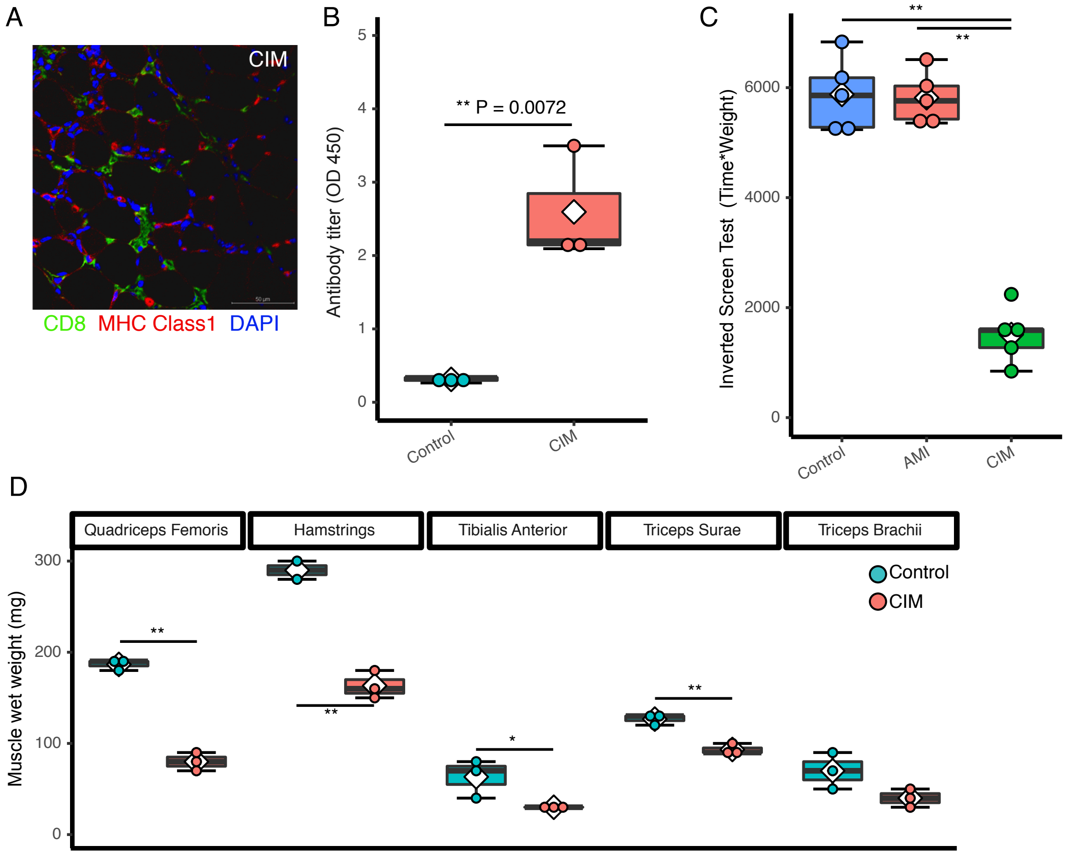

Supplementary Figure 1. Characteristics of CIM model mice. (A) Representative images of CD8- and MHC class 1-immunostained triceps surae. (B) Serum antibody titer to crude-myosin in CIM and control mice. (C-D) Results of the inverted screen test for assessment of limb grip strength test (C), and muscle wet weight at 14 days after first immunization(D). Quantitative data are shown as means as well as medians with IQRs and 1.5 times the IQR, and are displayed by dot plots and box and whisker plots. *P*-values were determined by the two-tailed Student's t-test or one-way ANOVA adjusted by the Holm method (**P* < 0.05, ***P* < 0.001).

Supplementary Figure 2

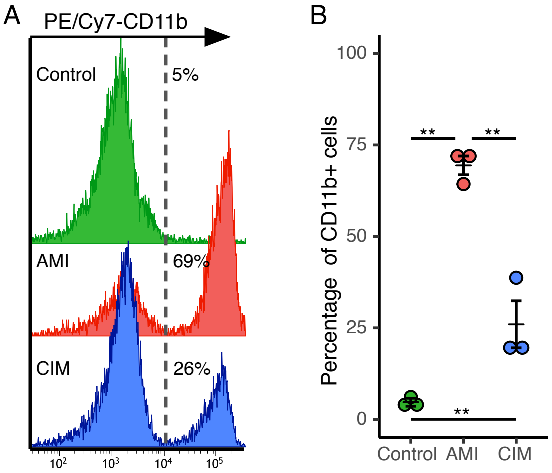

Supplementary Figure 2. Flow cytometric analysis of CD11b in mononuclear cells in muscle. (A)

Representative CD11b histograms from n = 3 replicates, and (B) quantification of percentages of CD11b+ cells.

Supplementary Figure 3

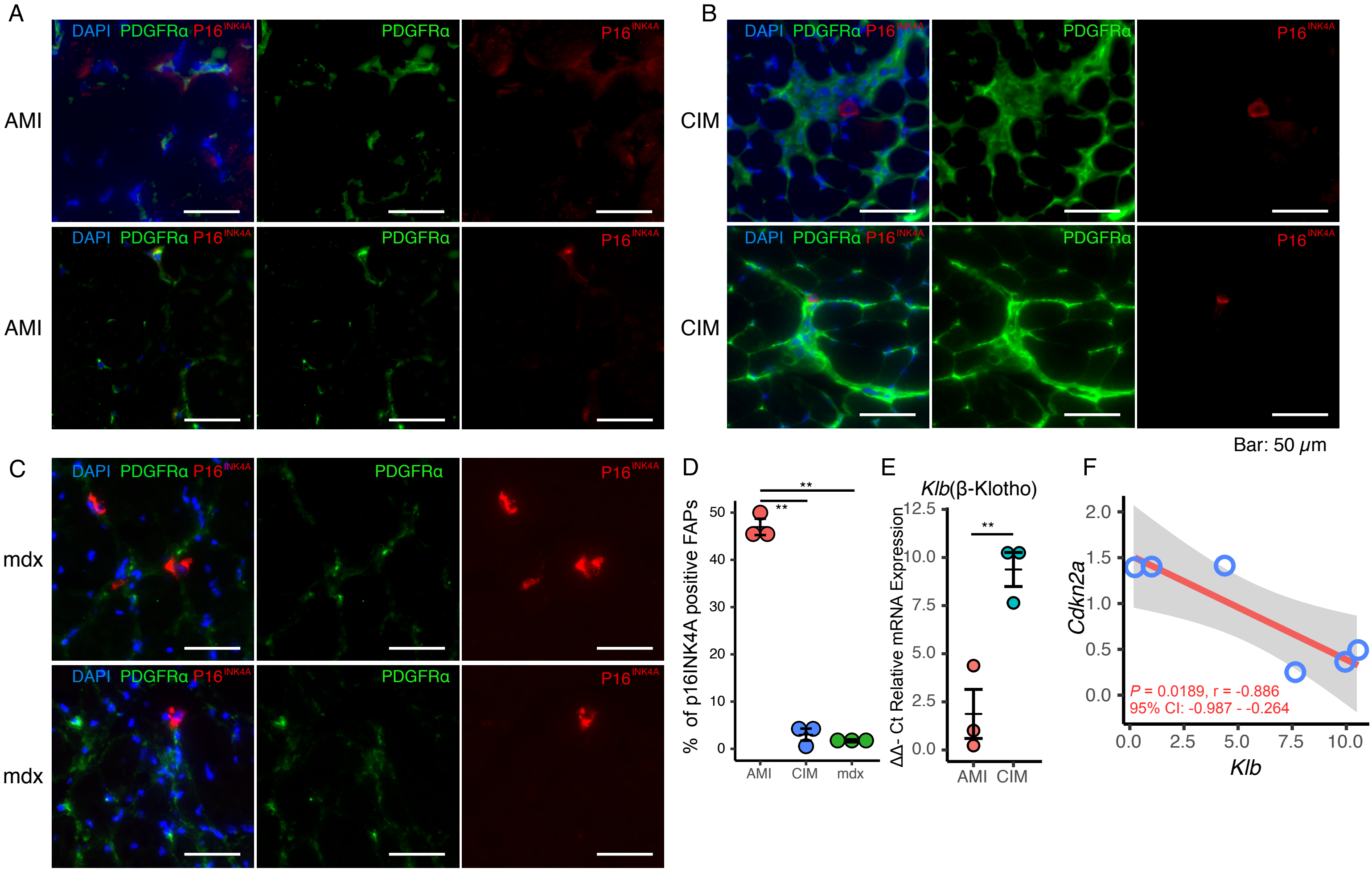

Supplementary Figure 3. Immunohistochemical analysis of p16INK4A expression in AMI, CIM, and mdx mice. (A-D) Representative images of PDGFRα- and p16INK4A-immunostained muscle (green and red, respectively) in AMI, CIM, and mdx mice (A-C), and quantitative data of the percentages of p16INK4A+ FAPs (D) in randomly chosen fields of view. (E and F) mRNA expression of Klb in FAPs isolated from AMI and CIM mice (n = 3 per group) (E), and correlation of Klb mRNA expression with Cdkn2a mRNA expression in FAPs isolated from AMI and CIM mice (F). Quantitative data are shown as means ± SEM (dot plot). *P*-values were determined by two-tailed Student's t-test (**P* < 0.05, ***P* < 0.001).

Supplementary Figure 4

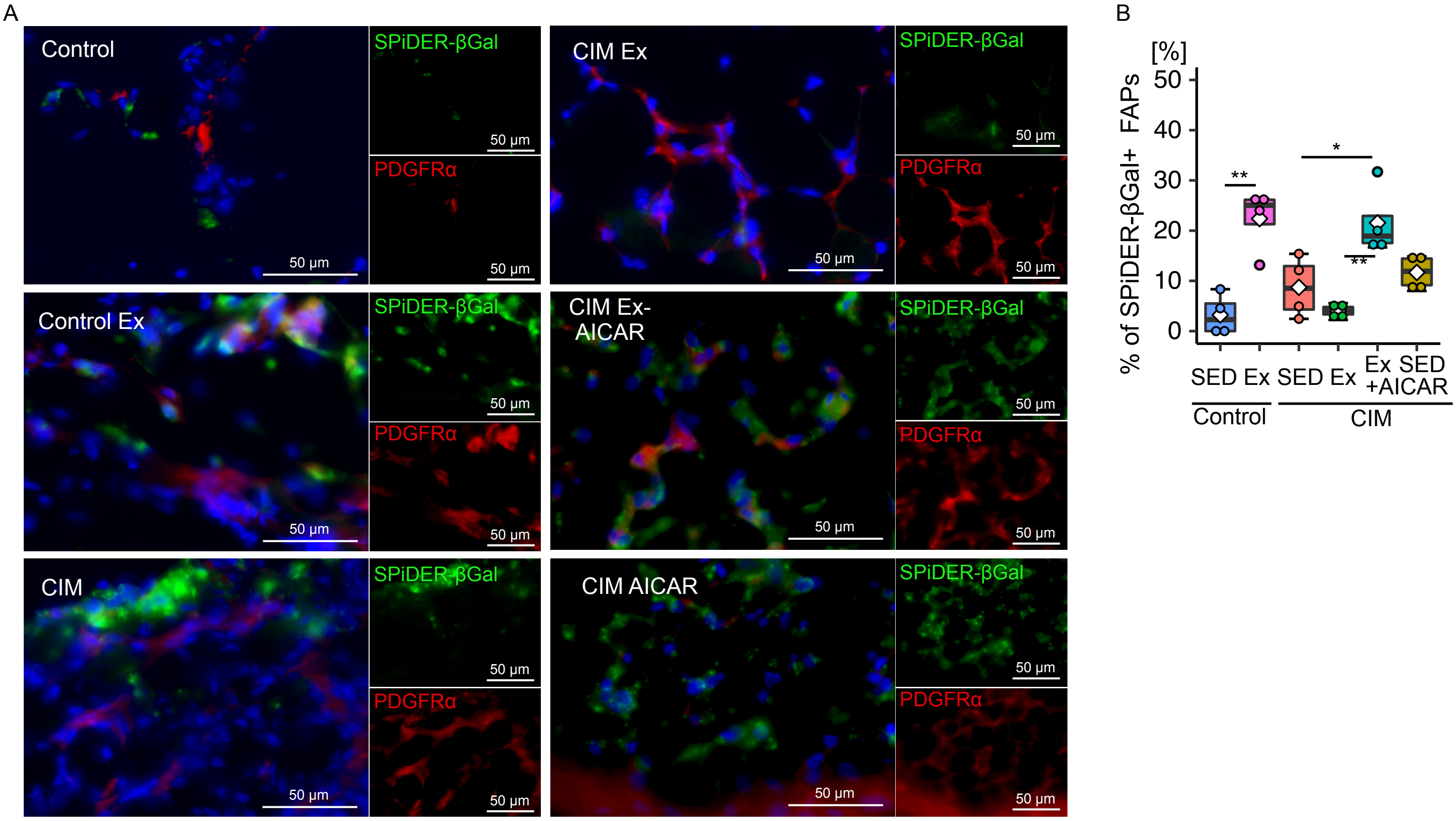

Supplementary Figure 4. SPiDER-βGal expression in PDGFRα-positive FAPs in exercised muscle.

(A and B) Representative images of SPiDER-βGal- and PDGFRα-immunostained muscle in control and CIM mice with or without exercise and AICAR treatment (A), and quantitative data of the SPiDER-βGal–positive FAPs(B).

Supplementary Figure 5

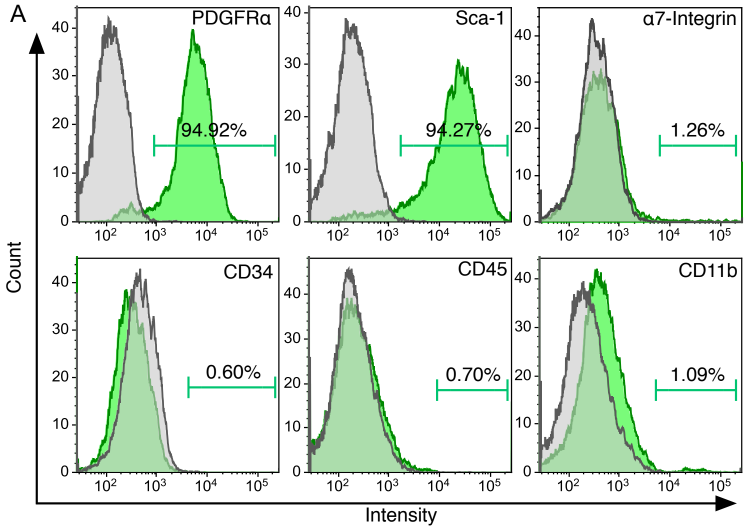

Supplementary Figure 5. Flow cytometric analysis of magnetic isolated Lin-, CD31-, α7-integrin- cultured cell. (A) Expressions of cell surface antigens PDGFRα, Sca-1, α7-integrin, CD34, CD45, and CD11b. Positive gates were set by analyzing negative control sample stained with isotype control or 2nd antibody only (gray area). The percentage of each cell population are shown in the panels.

Supplementary Figure 6

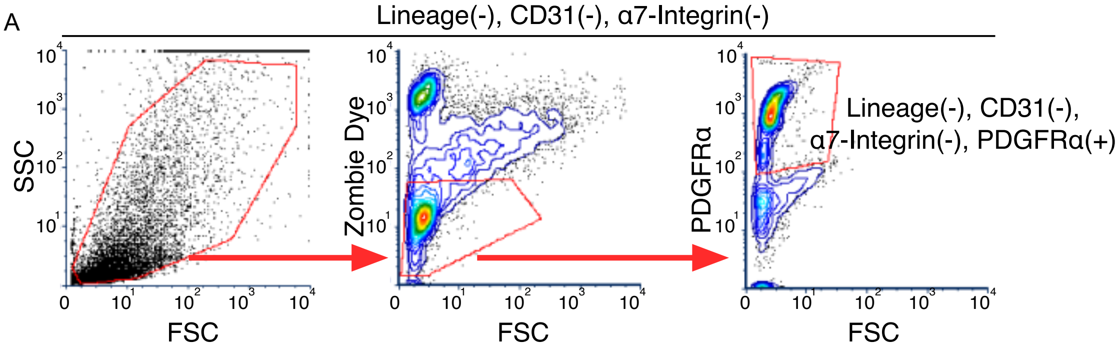

Supplementary Figure 6. Gating strategies used in flow cytometric analysis. (A) Magnetically isolated Lin-, CD31-, α7-integrin- cells were used. Zombie dye positive cells are gated out as dead cells. Zombie dye-, PDGFRα+ cells were used as FAPs.

Supplementary table 1
Primary antibodies used

Antibody	Clone	Conjugate(s)	Dulution	Source	Cat. No.
Magnetic cell sorting and Flow cytometry					
rabbit anti-PDGFRα	polyclonal	unconjugated	1:200	Santa Cruze Biotechnology	SC-338
rat anti-CD31	MEC13.1	biotin	1:200	Biolegend	102503
rat anti-integrin α7	3C12	biotin	1:20	Miltenyi Biotec	130-102-125
Lineage cell detection cocktail	rat anti-CD5	biotin	1:10	Miltenyi Biotec	130-092-613
	rat anti-CD11b				
	rat anti-CD45R				
	rat anti-7-4				
	rat anti-Gr-1				
	rat anti-Ter-119				
rat anti-CD140a	APA5	biotin, PE	1:200	Biolegend	135909, 135905
rabbit anti-p16INK4A	Polyclonal	unconjugated	1:200	ProteinTech	10883-1-AP
mouse anti-p53	1C12	Alexa Fluor 647	1:50	Cell Signaling	2533S
mouse anti-Bcl2	BCL/10C4	Alexa Fluor 488	1:80	Biolegend	633505
rat anti-IL-33	396118	Alexa Fluor 488	1:50	Novus Biologicals	IC3626G
mouse anti-phospho p38 MAPK(Thr180/Tyr182)	36/p38	PE/Cy7	1:5	BD Biosciences	560241
rabbit anti-phospho NF-κB p65 (Ser536)	93H1	Alexa Fluor 647	1:50	Cell Signaling	4887S
rat anti-CD45	30-F11	FITC	1:200	Biolegend	103107
rat anti-Sca-1	D7	PE/Cy7	1:200	Biolegend	108113
rat anti-CD34	RAM34	Alexa Fluor 700	1:200	eBioscience	56-0341-82
rat anti-CD11b	M1/70	PE/Cy7	1:80	Biolegend	101215
Immunohistochemistry and immunofluorescence					
rat anti-CD140a	APA5	unconjugated	1:50-200	Biolegend	135902
rabbit anti-collagen type I	Polyclonal	unconjugated	1:100	Abcam	ab34710
rabbit anti-laminin	Polyclonal	unconjugated	1:100	Abcam	ab11575
rabbit anti-p16INK4A	Polyclonal	unconjugated	1:100	ProteinTech	10883-1-AP
mouse anti-p53	1C12	Alexa Fluor 647	1:50	Cell Signaling	2533S
mouse anti-Bcl2	BCL/10C4	Alexa Fluor 488	1:50	Biolegend	633505
rat anti-IL-33	396118	Alexa Fluor 488	1:50	Novus Biologicals	IC3626G
rabbit anti-active caspase-3	Polyclonal	unconjugated	1:125	Promega	G7481
mouse anti-Myosin 4	MF20	Alexa Fluor 488	1:100	Invitrogen	53-6503-82
rat anti-CD8	53-6.7	unconjugated	1:80	Biolegend	100701
mouse anti-H-2Kd	SF1-1.1	biotin	1:80	Biolegend	116603

Secondary reagent used

Scondary Reagent	Conjugate(s)	Source
goat polyclonal anti-rabbit IgG	Alexa Fluor 488, Alexa Fluor 647	Jackson ImmunoReserch
goat polyclonal anti-rat IgG	Alexa Fluor 488, Alexa Fluor 647, Cy3	Jackson ImmunoReserch
Streptavidin	Alexa Fluor 647	Jackson ImmunoReserch

SupplementaryTable 2. Specific primer sequence used for real-time PCR

Gene	Forward	Reverse	Size	Accession Number
Cd274	5' GGAATTGTCTCAGAATGGTC 3'	5' GTAGTTGCTTCTAGGAAGGAG 3'	102	NM_021893.3
Pdcd1lg2	5' AAGACTGACAATCTTCCCTC 3'	5' CCTGAAAGTCATTAGGAGCC 3'	122	NM_021396.2
Cd47	5' TGCGGTTCAGCTCAACTACTG 3'	5' GCTTTGCGCCTCCACATTAC 3'	108	NM_010581.3
Cdkn2a	5' CGAACTCTTTCGGTCGTACCC 3'	5' CGAATCTGCACCGTAGTTGAGC 3'	89	NM_001040654.1
Trp53	5' AGCATCTTATCCGGGTGGAAG 3'	5' CCCATGCAGGAGCTATTACACA 3'	157	NM_011640.3
P21	5' GTACTTCCTCTGCCCTGCTG 3'	5' AGAAGACCAATCTGCGCTTG 3'	183	NM_007669.5
P19Arf	5' CGGAATCCTGGACCAGGTG 3'	5' ACCAGCGTGTCCAGGAAGC 3'	150	NM_009877.2
Il33	5' GGTGTGGATGGGAAGAAGCTG 3'	5' GAGGACTTTTTGTGAAGGACG 3'	155	NM_001164724.2
Klb	5' AGCCAATGGCATCGATGAC 3'	5' ACACGCAGGACTTCTGTTCT 3'	265	NM_031180.2
Tnfaip6	5' ACATGCAAAGGAGTGTGGTG 3'	5' GTGAATTCGCTGACCGTACTTG 3'	133	NM_009398.2
Tgfb1	5' GCCTGAGTGGCTGTCTTTTGA 3'	5' CACAAGAGCAGTGAGCGCTGAA 3'	101	NM_011577.2
Acta1	5' ACGCTGAAGTATCCGA 3'	5' CATTTTCTCCCGGTTGG 3'	162	NM_007392.3
Fst	5' AACCTACCGCAACGAATGTG 3'	5' TGGTCTGATCCACCACACAAG 3'	143	NM_001301373.1
Gapdh	5' AGGTCGGTGTGAACGGATTTG 3'	5' TGTAGACCATGTAGTTGAGGTCA 3'	123	NM_001289726.1
18S rRNA	5' GTAACCCGTTGAACCCCATT 3'	5' CCATCCAATCGGTAGTAGCG 3'	151	NR_003286

Supplementary Table 3. Primer sequence used for Trp53 mice genotyping

Primer name	Sequence
p53WTKO-Rv2	ACACCCAACACCATACCATGT
NP4	TGCTCGACGTTGTCACTGAAGC
LCB614(int-1)	GTTATGCATCCATACAGTACA
