# Supplementary material for: Exercise enhances skeletal muscle regeneration by promoting senescence in fibro-adipogenic progenitors
Source: Nat Commun. 2020 Feb 14;11:889. doi: 10.1038/s41467-020-14734-x (PMC7021787; doi:10.1038/s41467-020-14734-x)
Supplement: Supplementary file 2 — Reporting Summary [file 41467_2020_14734_MOESM2_ESM.pdf]

nature research

Corresponding author(s): Takako S. Chikenji

Last updated by author(s): Jan 1, 2020

Reporting Summary

Nature Research wishes to improve the reproducibility of the work that we publish. This form provides structure for consistency and transparency in reporting. For further information on Nature Research policies, see Authors & Referees and the Editorial Policy Checklist .

Statistics

For all statistical analyses, confirm that the following items are present in the figure legend, table legend, main text, or Methods section.

n/a	Confirmed	
☐	☒	The exact sample size (n) for each experimental group/condition, given as a discrete number and unit of measurement
☐	☒	A statement on whether measurements were taken from distinct samples or whether the same sample was measured repeatedly
☐	☒	The statistical test(s) used AND whether they are one- or two-sided<br>*Only common tests should be described solely by name; describe more complex techniques in the Methods section.*
☒	☐	A description of all covariates tested
☐	☒	A description of any assumptions or corrections, such as tests of normality and adjustment for multiple comparisons
☐	☒	A full description of the statistical parameters including central tendency (e.g. means) or other basic estimates (e.g. regression coefficient) AND variation (e.g. standard deviation) or associated estimates of uncertainty (e.g. confidence intervals)
☐	☒	For null hypothesis testing, the test statistic (e.g. F, t, r) with confidence intervals, effect sizes, degrees of freedom and P value noted<br>*Give P values as exact values whenever suitable.*
☒	☐	For Bayesian analysis, information on the choice of priors and Markov chain Monte Carlo settings
☐	☒	For hierarchical and complex designs, identification of the appropriate level for tests and full reporting of outcomes
☐	☒	Estimates of effect sizes (e.g. Cohen's d, Pearson's r), indicating how they were calculated

Our web collection on statistics for biologists contains articles on many of the points above.

Software and code

Policy information about availability of computer code

Data collection	PrimePCR gene expression levels were calculated by the PrimerArray Analysis Tool Ver. 2.2. Flow cytometry data collection and analysis were conducted using FACSDivaTM (BD Biosciences) and FCS Express 6 (De Novo software). Immunocytochemistrical and immunohistochemical data collection and analysis were conducted confocal laser scanning microscope (Nikon/A1; Nikon, Tokyo, Japan) or fluorescence microscope BZ-X700 (Keyence Corp., Osaka, Japan).
Data analysis	Quantitative data are shown as means as well as medians with interquartile ranges (IQRs) and 1.5 times the IQR, and displayed by dot plots and box and whisker plots using ggplot2, which is a plotting system for R based on The Grammar of Graphics (The R Foundation for Statistical Computing, Vienna, Austria). Heat map, k-means clustering, and principal component analyses were performed by FactoMineR and factoextra, which are R packages. P-values for multiple comparisons were adjusted by the Holm method. Statistical analyses were performed using EZR, which is a graphical user interface for R.

For manuscripts utilizing custom algorithms or software that are central to the research but not yet described in published literature, software must be made available to editors/reviewers. We strongly encourage code deposition in a community repository (e.g. GitHub). See the Nature Research guidelines for submitting code & software for further information.

Data

Policy information about availability of data

All manuscripts must include a data availability statement. This statement should provide the following information, where applicable:

- Accession codes, unique identifiers, or web links for publicly available datasets
- A list of figures that have associated raw data
- A description of any restrictions on data availability

Data and materials not indicated in this manuscript are available from the corresponding author.

Field-specific reporting

Please select the one below that is the best fit for your research. If you are not sure, read the appropriate sections before making your selection.

☒ Life sciences ☐ Behavioural & social sciences ☐ Ecological, evolutionary & environmental sciences

For a reference copy of the document with all sections, see nature.com/documents/nr-reporting-summary-flat.pdf

Life sciences study design

All studies must disclose on these points even when the disclosure is negative.

Sample size	Power calculations were not performed to predetermine the sample size.
Data exclusions	Animals were excluded from the study only if their health status was compromised, for instance if they had visible wounds due to fighting.
Replication	All experiments were replicated in a at least two independent experiments performed under identical conditions.
Randomization	We did not use any specific method of randomization to determine how animals were allocated to experimental groups.
Blinding	The investigators were not blinded to allocations during experiments or outcome assessments, though they were blinded during the behavioral assessment.

Reporting for specific materials, systems and methods

We require information from authors about some types of materials, experimental systems and methods used in many studies. Here, indicate whether each material, system or method listed is relevant to your study. If you are not sure if a list item applies to your research, read the appropriate section before selecting a response.

Materials & experimental systems

n/a	Involved in the study	
☐	☒	Antibodies
☐	☒	Eukaryotic cell lines
☒	☐	Palaeontology
☐	☒	Animals and other organisms
☒	☐	Human research participants
☒	☐	Clinical data

Methods

n/a	Involved in the study	
☒	☐	ChIP-seq
☐	☒	Flow cytometry
☒	☐	MRI-based neuroimaging

Antibodies

Antibodies used

Antibody/Clone/Conjugate(s)/Dulution/Source/Cat. No.
rabbit anti-PDGFRα polyclonal unconjugated 1:200 Santa Cruze Biotechnology SC-338
rat anti-CD31 MEC13.1 biotin 1:200 Biolegend 102503
rat anti-integrin α7 3C13 biotin 1:20 Miltenyi Biotec 130-102-125
Lineage cell detection cocktail biotin 1:10 Miltenyi Biotec 130-092-613
rat anti-CD140a APA5 biotin, PE 1:200 Biolegend 135909, 135905
rabbit anti-p16INK4A Polyclonal unconjugated 1:200 ProteinTech 10883-1-AP
mouse anti-p53 1C12 Alexa Fluor 647 1:50 Cell Signaling 2533S
mouse anti-Bcl2 BCL/10C4 Alexa Fluor 488 1:80 Biolegend 633505
rat anti-IL-33 396118 Alexa Fluor 488 1:50 Novus Biologicals IC3626G
mouse anti-phospho p38 MAPK(Thr180/Tyr182) 36/p38 PE/Cy7 1:5 BD Biosciences 560241
rabbit anti-phospho NF-κB p65 (Ser536) 93H1 Alexa Fluor 647 1:50 Cell Signaling 4887S
rat anti-CD45 30-F11 FITC 1:200 Biolegend 103107
rat anti-Sca-1 D7 PE/Cy7 1:200 Biolegend 108113
rat anti-CD34 RAM34 Alexa Fluor 700 1:200 eBioscience 56-0341-82
rat anti-CD11b M1/70 PE/Cy7 1:80 Biolegend 101215
rat anti-CD140a APA5 unconjugated 1:50-200 Biolegend 135902
rabbit anti-collagen type I Polyclonal unconjugated 1:100 Abcam ab34710
rabbit anti-laminin Polyclonal unconjugated 1:100 Abcam ab11575
rabbit anti-p16INK4A Polyclonal unconjugated 1:100 ProteinTech 10883-1-AP
mouse anti-p53 1C12 Alexa Fluor 647 1:50 Cell Signaling 2533S
mouse anti-Bcl2 BCL/10C4 Alexa Fluor 488 1:50 Biolegend 633505

	rat anti-IL-33 396118 Alexa Fluor 488 1:50 Novus Biologicals IC3626G<br>rabbit anti-active caspase-3 Polyclonal unconjugated 1:125 Promega G7481<br>mouse anti-Myosin 4 MF20 Alexa Fluor 488 1:100 Invitrogen 53-6503-82<br>rat anti-CD8 53-6.7 unconjugated 1:80 Biolegend 100701<br>mouse anti-H-2Kd SF1-1.1 biotin 1:80 Biolegend 116603
Validation	rabbit anti-PDGFRα https://www.scbt.com/ja/p/pdgfr-alpha-antibody-c-20<br>rat anti-CD31 https://www.biolegend.com/en-us/products/biotin-anti-mouse-cd31-antibody-376<br>rat anti-integrin α7 https://www.miltenyibiotec.com/JP-en/products/macs-flow-cytometry/antibodies/primary-antibodies/anti-integrin-a7-antibodies-mouse-3c12-1-10.html<br>Lineage cell detection cocktail https://www.miltenyibiotec.com/FR-en/products/macs-flow-cytometry/kits-and-support-reagents/phenotyping-assays/lineage-cell-detection-cocktail-biotin-mouse.html<br>rat anti-CD140a https://www.biolegend.com/en-us/products/pe-anti-mouse-cd140a-antibody-6253<br>rabbit anti-p16INK4A https://www.ptglab.com/Products/P16,P19-Antibody-10883-1-AP.htm<br>mouse anti-p53 https://www.cellsignal.com/products/antibody-conjugates/p53-1c12-mouse-mab-alexa-fluor-647-conjugate/2533<br>mouse anti-Bcl2 https://www.biolegend.com/en-us/products/alexa-fluor-488-anti-bcl-2-antibody-6346<br>rat anti-IL-33 https://www.rndsystems.com/products/mouse-il-33-alexa-fluor-488-conjugated-antibody-396118_ic3626g<br>mouse anti-phospho p38 MAPK(Thr180/Tyr182) https://www.bdbiosciences.com/eu/applications/research/intracellular-flow/intracellular-antibodies-and-isotype-controls/anti-rat-antibodies/pe-cy7-mouse-anti-p38-mapk-pt180py182-36p38-pt180py182/p/560241<br>rabbit anti-phospho NF-κB p65 (Ser536) https://www.cellsignal.jp/products/antibody-conjugates/phospho-nf-kb-p65-ser536-93h1-rabbit-mab-alexa-fluor-647-conjugate/4887<br>rat anti-CD45 https://www.biolegend.com/en-us/products/fitc-anti-mouse-cd45-antibody-99<br>rat anti-Sca-1 https://www.biolegend.com/en-us/products/pe-cy7-anti-mouse-ly-6a-e-sca-1-antibody-3137<br>rat anti-CD34 https://www.thermofisher.com/antibody/product/CD34-Antibody-clone-RAM34-Monoclonal/56-0341-82<br>rat anti-CD11b https://www.biolegend.com/en-us/products/pe-cy7-anti-mouse-human-cd11b-antibody-1921<br>rat anti-CD140a https://www.biolegend.com/en-us/products/purified-anti-mouse-cd140a-antibody-6251<br>rabbit anti-collagen type I https://www.abcam.co.jp/collagen-i-antibody-ab34710.html<br>rabbit anti-laminin https://www.abcam.co.jp/laminin-antibody-ab11575.html<br>rabbit anti-p16INK4A https://www.ptglab.com/Products/P16,P19-Antibody-10883-1-AP.htm<br>mouse anti-p53 https://www.cellsignal.com/products/antibody-conjugates/p53-1c12-mouse-mab-alexa-fluor-647-conjugate/2533<br>mouse anti-Bcl2 https://www.biolegend.com/en-gb/products/alexa-fluor-488-anti-bcl-2-antibody-6346<br>rat anti-IL-33 https://www.rndsystems.com/products/mouse-il-33-alexa-fluor-488-conjugated-antibody-396118_ic3626g<br>rabbit anti-active caspase-3 https://www.promega.jp/products/cell-health-assays/apoptosis-assays/anti_active-caspase_3-pab/<br>mouse anti-Myosin 4 https://www.thermofisher.com/antibody/product/Myosin-4-Antibody-clone-MF20-Monoclonal/53-6503-82<br>rat anti-CD8 https://www.biolegend.com/en-us/products/purified-anti-mouse-cd8a-antibody-157<br>mouse anti-H-2Kd https://www.novusbio.com/products/h-2kd-antibody-sf1-11_nb100-77621

Eukaryotic cell lines

Policy information about cell lines

Cell line source(s)	RAW264.7 were purchased from American Type Culture Collection (ATCC)<br>C2C12 myoblasts were purchased from European Collection of Authenticated Cell Cultures (ECACC).
Authentication	As described by ATCC and ECACC
Mycoplasma contamination	Cell lines were tested for mycoplasma using the e-Myco™ Mycoplasma PCR Detection Kit (iNtRON Biotechnology, Inc., Seongnam-si, South Korea).
Commonly misidentified lines<br>(See ICLAC register)	n/a

Animals and other organisms

Policy information about studies involving animals; ARRIVE guidelines recommended for reporting animal research

Laboratory animals	Female BALC/c and C57BL/6 mice (age >6 weeks; Sankyo Lab Service, Tokyo, Japan)<br>B6.Cg-Trp53<tm1Sia>/Rbrc mice (C57BL-p53+/-) (Accession No. CDB 0001K) were provided by the RIKEN BioResource Center (Ibaragi, Japan)
Wild animals	This study did not use wild animals.
Field-collected samples	This study did not use field-collected samples.

Ethics oversight | The Committee of the Animal Experimentation Center of the Sapporo Medical University School of Medicine approved all animal protocols.

Note that full information on the approval of the study protocol must also be provided in the manuscript.

Flow Cytometry

Plots

Confirm that:

- [x] The axis labels state the marker and fluorochrome used (e.g. CD4-FITC).
- [x] The axis scales are clearly visible. Include numbers along axes only for bottom left plot of group (a 'group' is an analysis of identical markers).
- [x] All plots are contour plots with outliers or pseudocolor plots.
- [x] A numerical value for number of cells or percentage (with statistics) is provided.

Methodology

Sample preparation	Triceps surae were carefully dissected to remove attached tendons, nerves, blood vessels, and fat tissue. Muscles were dissociated mechanically and digested in 0.2% collagenase type 2 (Worthington Biochemical Corporation; New Jersey, USA) and 2.5 mM CaCl2 in PBS for 60 min at 37°C. Digested muscles were passed through an 18-gauge needle several times and further digested for 30 min at 37°C. Muscle slurries were filtered through a 100-μm cell strainer (EASYstrainerTM Cell; Greiner Bio-One, Kremsmuenster, Austria), and through a 40-μm cell strainer (Greiner Bio-One). Erythrocytes were eliminated by treating the cells with RBC lysis solution (QIAGEN, Hilden, Germany). Magnetic isolated Lin-/CD31-/α7-integrin- cells were stained with different antibodies and analyzed for multi-color flow cytometry.<br>The isolated cells were stained with Zombie VioletTM Dye (Biolegend) in PBS at a 1:100 dilution for 15 min, washed, and fixed and permeabilized with the PerFix-nc Kit (Beckman Coulter) for intercellular cytokines, or the PerFix-EXPOSE Phospho Epitope Exposure Kit (Beckman Coulter) for the detection of phosphorylated intracellular antigens.
Instrument	Flow cytometry analysis was performed using FACSCantoTM II (BD Biosciences) equipped with 405-nm, 488-nm, and 633-nm lasers.
Software	Data collection and analysis were conducted using FACSDivaTM (BD Biosciences) and FCS Express 6 (De Novo software).
Cell population abundance	Flow cytometry analysis was performed on specific population after magnetic cell isolation.
Gating strategy	Gating strategies for cell populations is shown in supplementary figure 6.

- [x] Tick this box to confirm that a figure exemplifying the gating strategy is provided in the Supplementary Information.
